# Cultural participation and self-reported health: a longitudinal population-based study from Sweden

**DOI:** 10.1080/02813432.2026.2739500

**Published:** 2026-10-29

**Authors:** Filip Jansåker, Kristina Sundquist, Lars Olov Bygren, Sven-Erik Johansson

**Affiliations:** a Center for Primary Health Care Research, Department of Clinical Sciences Malmö, Lund University, Sweden; b University Clinic Primary Care, Skåne University Hospital, Sweden; c Center for Community-based Healthcare Research and Education (CoHRE), Department of Functional Pathology, School of Medicine, Shimane University, Japan; d Community Medicine and Rehabilitation, Umeå University, Umeå, Sweden; e Department of Biosciences and Nutrition, Karolinska Institutet, Huddinge, Sweden

**Keywords:** Cultural events, cultural participation, epidemiology, health, self-reported health

## Abstract

**Objective:**

Culture is an essential part of human life. This study aimed to examine the association between cultural participation and self-reported health.

**Methods:**

A longitudinal cohort study of a random sample (3311 participants) of the Swedish population aged 20–67 years at baseline was performed. Data were collected for all participants on three occasions (1982/83, 1990/91, and 1998/99) including cultural participation (visiting cinema, theatre, concert, art museum, other museums, and sermon), covariates (age, sex, region of residency, cohabitation status, level of education, physical activity, and smoking status), and outcome (self-reported health). To analyze the association between cultural participation level and impaired self-reported health with time-varying covariates, Generalized Estimating Equations were used to estimate odds ratios with 95% confidence intervals. A sub-analysis on data from the first two occasions and two sensitivity analyses were conducted.

**Results:**

The results suggested a graded association between cultural participation and self-reported health, i.e. the lower the exposure to cultural activities the poorer the self-reported health. In the full model, participants with low cultural participation level had higher odds (odds ratio: 1.91; 95% confidence intervals: 1.62–2.26) of impaired self-reported health than participants reporting high cultural participation level, adjusting for potential confounders. The sub- and sensitivity analyses yielded similar findings.

**Conclusion:**

The findings of this longitudinal study suggest that cultural participation may play an important role in promoting good health and wellbeing. This knowledge could be particularly valuable for policymakers and primary healthcare providers seeking to promote greater access to and participation in cultural activities within society.

## Background

Culture is an essential component in human development and life and the experience of art, through various forms such as museums, music, and theater, is not merely an enjoyment in life but also seems to have a pivotal role for maintaining good health [1]. Already in 1934, the philosopher John Dewey [2] elaborated on the positive experience of art, introducing how enjoyment of art can have profound effects on wellbeing. For use in public health promotion this might be crucial, as cultural participation could have health promoting effects well matching others, such as smoking cessation [3] and physical activity [4].

The societal actions for promoting health by cultural participation has increased over time [5] and the scientific evidence that cultural participation affects health and mortality has been accumulating. Already two decades ago, our research group found attendance of art events to be inversely associated with all-cause mortality [6]; this has been subsequently replicated in other studies [7–9]. Other studies have found associations between cultural participation and chronic pain prevention in elderly [10], depression [11,12], and cardiovascular disease risk and mortality [13–16]. High cultural participation is also associated with a lower number of primary healthcare visits [17] and seems to promote well-being, life satisfaction, and self-reported health (SRH) [11,12,18–21]. In addition, SRH is an important measure in epidemiological studies and established predictor for morbidity and mortality [22], and some studies have found that SRH could be positively affected by cultural participation.

For example, studies on adults from Finland and Denmark reported positive associations between cultural participation and SRH [12,19], and a study from Norway found that adolescents frequently participating in cultural activities reported better health compared to inactive peers [18]. However, studies from Poland [23] and Switzerland [24] found no significant associations between cultural participation and SRH. In a 2001 study of ours, we found that the relationship between cultural participation and SRH remained if high cultural participation was maintained over time. In addition, increasing the cultural participation level during the follow-up was also positive for SRH, while giving up cultural participation was followed by impaired-SRH [20]. That study did, however, not account for variations in the covariates over time, which will be accounted for in the present study.

Several explanations behind the inconsistencies in previous research may exist, such as differences in the measurement of SRH, covariates, setting, and design. In the present study, we adopted a longitudinal approach where we adjusted for time-varying covariates to examine the association between cultural participation (measured as the frequency of visiting cinemas, theatres, concerts, art museums, other museums, and sermons) and SRH in a simple random sample of the Swedish population [25], considering the potential changes of all included confounders (e.g. smoking, physical activity) over time. In addition, the mechanisms through which cultural participation may affect health in humans are complex and not entirely understood, and could involve both biological, behavioral, psychological, and social factors [26] and their interactions/potential effect modifications.

The aim was to analyze the longitudinal association between cultural participation and SRH, adjusting for important confounders and examining potential effect modifiers, such as lifestyle and sociodemographic factors. To our knowledge, no previous study has adopted this longitudinal approach, with time-varying covariates collected at three measurements, to examine this association. Based on previous literature [11,12,18–21], our hypothesis was that cultural participation has a health promoting effect when analyzed longitudinally.

## Material and methods

### Study design and setting

This longitudinal study included 3311 participants with three measurements of cultural participation (1982/83, 1990/91, and 1998/99) and self-reported health (SRH); i.e. the 3311 individuals were selected based on participation in each of the three measurements and having complete data for cultural participation and SRH. Thus, the longitudinal approach was based on the same individuals being repeatedly observed over the study period. The study was conducted at Lund University, Sweden. The STROBE statement-checklist for cohort studies was considered.

## Data source

The database used to identify the study population, outcome, and all variables examined was the Statistics on Income and Living Conditions [25], managed by the governmental authority on national statistics, Statistics Sweden (Swedish: “Statistikmyndigheten”, SCB). The name of this database in Swedish is “Undersökningarna av levnadsförhållanden” (throughout this paper: ULF). ULF was based on a simple random sample of individuals aged 16–84 years from the Swedish population and data were primarily collected through personal in-home interviews before 2008, after which telephone interviews became the most common practice.

### Ascertainment of study population

The study population was a simple random sample of the Swedish population invited to partake in repeated measurements in the national survey ULF [25]. To fully take into account the potentially time varying nature of our variables, we only included participants who had partaken in all three measurements. A total of 3311 men and women took part in all three measurements and were included in the study [9], which were 83% of those (*n* = 3983) who had taken part in the first two measurements. In the dataset that was available to us, however, it was not possible for us to assess the number of participants who only partook in the first measurement. This is because the group that partook in only one measurement consists of a much larger sample and only a sub-group of these were invited to partake in repeated measurements over time.

### Ascertainment of the outcome

SRH was measured at 1982/83 (baseline), 1990/91 and 1998/99 [25]. “Impaired-SRH” (=1, in the analyses) was defined as SRH status other than “Good-SRH” (=0, in the analyses). The SRH was based on the following question asked to the participants in ULF^25^ (translated from Swedish): “How would you assess your general health condition?”. The first two measurement points were based on a 3-graded ordinal scale. SRH being reported as “good” was defined as Good-SRH and SRH being reported as “poor” or “somewhere in-between [poor and good]” was defined as Impaired-SRH. In the third measurement, the questions on SRH had been changed from the previous 3-graded to a 5-graded ordinal scale. For this reason, we conducted a sub-analysis of those who had partaken in the first two measurements only (see below). For these analyses, we defined Good-SRH as SRH being reported as “very good” or “good” and Impaired-SRH as SRH being reported as “very poor”, “poor” or “fair”.

### Ascertainment of the main predictor

The main predictor was defined as the level of attending cultural events (cultural participation) at the three measurement points. The level of cultural participation was based on an index computed from several self-reported cultural participation measured in ULF [25], which was used for all three measurements in this study. The forms of cultural participation that were included in the index were based on previous research and established definitions on concepts of cultural events, which is quite broad and include a variety of cultural activities, such as art, musical activities, and religious activities (which also have been found to affect health) [21,27,28]. Therefore, we defined the main predictor variable in the present study as frequency of visiting (from never to every week or more often) cinemas, theatres, concerts, art museums, other museums, and sermons. We chose to include sermons in the cultural participation variable because religion is an important part of many cultures and have been recommended to include in measures of cultural participation [29]. Based on scores from a principal component analysis (polychoric correlation coefficient for ordinal data was used) with one factor, an index on the level of cultural participation was constructed. This index was constructed based on data from the first measurement on cultural participation (1982/83) [6], has been used for all three measurements in previous studies [9,13], and the questions it was based on were used similarly at all measurements. The variable was categorized approximately into the lowest (25%), the middle two (50%), and highest (25%) quartiles of cultural participation based on the index score and labeled throughout the study as Low, Middle, and High cultural participation. High cultural participation was defined as the reference group in our analyses (Odds Ratio, OR = 1).

### Ascertainment of the covariates

All covariates were longitudinally measured at the same time as the main predictor at the measurement points in ULF [25]. The covariates were chosen based on previous research [18–20] and included in order to adjust for potential confounding.

- Sex: Categorized as ‘man’ or ‘woman’ (the only variable that was not time varying, as no changes were observed between the measurements).
- Age: Continuous in the analysis. Age at baseline ranged from 20 to 67 years.
- Education: Self-reported and categorized into three groups ‘low’ (≤9 years of school, i.e., compulsory schooling or less), ‘medium’ (10–12 years of school, i.e., partial or complete high school education), and ‘high’ (≥13 years of school, i.e., at least one year of higher education).
- Cohabitation status: Self-reported and categorized as ‘married/cohabiting’ or ‘single living’.
- Region of residency: Categorized into ‘the three largest cities’ (Malmö, Göteborg, and Stockholm), ‘middle-sized towns’, and ‘other regions’.
- Current daily (tobacco) smoking: Self-reported and categorized as ‘yes’ or ‘no’.
- Physical exercise: Self-reported and categorized as ‘inactive’, ‘occasionally active’ or ‘actively exercising at least once a week’ either indoors or outdoors. It was dichotomized as ‘inactive/occasionally active’ vs. ‘active at least once a week’.

### Missing data

The selection of participants for our analytic sample had no missing data for the outcome and main predictor variables. However, missing data (<1%) existed for some covariates in ULF and participants with missing data were included in the models after imputations. Data was complete for the variables sex, age, cohabitation status, and region of residency. The partial non-response in the other covariates (i.e. education, current daily smoking, and physical activity) was less than one percent. These values were substituted by multiple imputation (MI), a method which is considered to be little biased [30].

### Statistical method

Descriptive statistics on the study population by interview year and cultural participation were calculated and presented. To assess the longitudinal association between cultural participation and SRH using time-varying covariates, all models were analyzed by a population average model with time-varying covariates and MI. For MI, we used procedure ‘MI’ in STATA and missing values were replaced with one set of plausible values, keeping the correlation structure in the data [31]. We used Generalized Estimating Equations (GEE) [32] and a population average model with an exchangeable correlation matrix with time-varying covariates to assess the association between cultural participation level and Impaired-SRH. GEE^32^ and alternating logistic regression (ALR) [33] often give similar results in the analysis of large samples, as in this study. A main advantage of GEE is the unbiased estimation of regression coefficients even at possible misspecification of the correlation structure. Therefore, we used GEE in the main analyses.

The first assessment was conducted in 1982/1983, the second in 1990/1991, and a third assessment included the years 1998/99. After imputation, GEE with time-varying covariates was applied to estimate ORs with 95% confidence intervals (CIs) in three models: A crude model, a model adjusted for sex and age, and a full model (adjusted for all covariates). Prevalence rates of Impaired-SRH (%) for each measurement and ORs for Impaired-SRH associated with the covariates used in the adjustments are also presented. STATA (StataCorp. 2019. *Stata Statistical Software: Release 16*. College Station, TX: StataCorp LLC.) was used for the descriptive analysis and GEE models, which were estimated with a binomial distribution, a logit link, and robust standard errors. A two-tailed p-value of <0.05 was defined as statistical significance. To examine whether the association between cultural participation level and Impaired-SRH varied by the covariates (e.g. physical activity, smoking status, and sociodemographic factors), interaction tests were conducted between cultural participation and each of the included covariates—no interactions were found (data not shown in tables). When judging the interactions, we calculated Bonferroni-adjusted [34] p-values in order to avoid spurious significant interactions. Due to the change from a 3-graded (1982/83 and 1990/91) to a 5-graded (1998/99) scale on SRH, we conducted a sub-analysis (full model by GEE) that only included data from responses based on the 3-graded scale to assess whether the associations remained.

We also conducted a sensitivity analysis (full model by GEE), which did not include the covariates current daily (tobacco) smoking and physical exercise in the adjustments, as these variables could act as both confounders or potential mediators in the association between cultural participation level and Impaired-SRH. An additional sensitivity analysis was conducted using ALR to estimate ORs with 95% CIs, given that ALR is considered to be an appropriate alternative to GEE when there is a binary response. This is because GEE might be biased as Pearson’s residuals are used to determine the correlation matrices, which sometimes is less appropriate when the outcome is binary [33]. SAS software (SAS Institute Inc. 2013. SAS^®^ 9.4 Statements: Reference. Cary, NC: SAS Institute Inc.) was used for the ALR models.

## Results

Table 1 includes the descriptive data, based on data from the 3311 participants, with three measurements, by year and level of cultural participation. The mean age was 39.8 years at the first measurement, ranging from 20 to 67 years. Some differences between the measurements in the distribution of the covariates seemed to exist, which indicates that the models need to take the time varying nature of the data into account.

**Table 1.** Distribution of the survey-based variables by year of interview (measurement) and cultural participation level (3311 participants, who completed all three measurements).

	First measurement (1982/83)			Second measurement (1990/91)			Third measurement (1998/99)		
	Cultural participation level [1]			Cultural participation level [1]			Cultural participation level [1]		
Variables	Low	Middle	High	Low	Middle	High	Low	Middle	High
Age (mean), years	42.6	39.3	38.2	49.8	47.3	47.0	62.6	54.1	53.5
Sex (%)									
Man	51.0	48.8	43.3	54.5	47.7	42.4	52.5	48.8	42.5
Woman	49.0	51.2	56.7	45.5	52.3	57.6	47.5	51.2	57.5
Education (%)									
≤ 9 years	85.3	72.7	43.8	81.8	68.6	38.4	86.2	66.8	34.7
10–12 years	12.6	20.7	34.9	16.4	23.1	34.3	11.3	24.7	37.4
>12 years	2.1	6.6	21.3	1.8	8.3	27.3	2.5	8.5	27.9
Cohabitation status (%)									
Married/cohabiting	78.7	76.0	62.8	76.6	78.7	76.7	63.7	75.1	77.0
Single living	21.3	24.0	37.2	23.4	21.3	23.3	36.3	24.9	23.0
Region of residency (%)									
Large cities	23.4	28.9	44.3	23.3	28.4	43.5	22.1	29.5	39.5
Middle-sized towns	34.4	31.0	27.8	34.9	35.9	33.3	36.6	36.6	35.2
All others	42.2	40.1	27.9	41.8	35.7	23.2	41.3	33.9	25.3
Daily smoking (%)									
Yes	38.9	30.5	26.2	33.1	26.6	21.8	24.0	20.8	14.2
No	61.1	69.5	73.8	66.9	73.4	78.2	76.0	79.2	85.8
Physical exercise (%)									
Inactive/occasionally	78.1	58.3	40.0	78.9	65.8	50.6	83.4	66.0	50.9
At least once a week	21.9	41.7	60.0	21.1	34.2	49.4	16.6	34.0	49.1

^1^Based on an index score from a principal component analysis (including the variables: visiting cinema, theatre, concert, art museum, other museums, and sermon) on complete cases. The participants were approximately categorized into four quartiles (the two middle quartiles were combined), with following levels: Low (≈25%, lowest quartile), Middle (≈50%, middle two quartiles), and High (≈25%, highest quartile). Percentages sum up to 100 % by Year and Cultural participation level, column-wise per variable.

Table 2 shows the prevalence of Impaired-SRH at the three measurements as well as Model 2, adjusted for sex and age, and Model 3, full model. In general, the prevalence of Impaired-SRH increased over time (i.e. with advanced age). This age-dependent decline in SRH was seen across all covariates. Participants with male sex, living in large cities and conducting physical exercise at least once a week had low prevalences of Impaired-SRH throughout the three measurements, while those reporting daily (tobacco) smoking and single living had high prevalences of Impaired-SRH. The association between cultural participation and SRH indicated a gradient, i.e. the lower the cultural participation level the higher the odds of Impaired-SRH. Table 3 shows that in the crude model, compared with high cultural participation level, low cultural participation level was associated with an OR for Impaired-SRH of 2.39 (95% CI, 2.06–2.77) and middle cultural participation level with an OR of 1.57 (95% CI, 1.38–1.79). The corresponding ORs were 2.30 (95% CI, 1.97–2.69) and 1.61 (95% CI, 1.41–1.85) in the age and sex adjusted model, and decreased to 1.91 (95% CI, 1.62–2.26) and 1.45 (1.25–1.67) in the full model.

**Table 2.** The annual prevalence (%) of impaired self-reported health (SRH) and the association between cultural participation level, covariates, and Impaired-SRH.

Covariates	Prevalence (%) of Impaired-SRH			Full model
	Year 1982/83	Year 1990/91	Year 1998/99	**OR** [**2**] **(CI)**
Impaired-SRH, totals	15.7	22.7	28.4	
Cultural participation^3^				
Low (≈25%)	22.8	32.1	46.4	1.91 (1.62–2.26)
Middle (≈50%)	15.0	22.2	27.2	1.45 (1.25–1.67)
High (≈25%)	10.4	14.6	15.2	1 (ref.)
Sex				
Man	14.8	20.4	25.9	1 (ref.)
Woman	16.6	24.8	30.6	1.23 (1.08–1.40) [4]
Education (years)				
≤9	19.0	27.5	33.6	1.48 (1.17–1.57)
10–12	9.2	15.0	20.7	1.19 (0.93–1.53)
>12	6.9	12.5	17.2	1 (ref.)
Cohabitation status				
Married/cohabiting	15.8	21.2	24.1	1 (ref.)
Single living	15.5	27.7	39.9	1.38 (1.22–1.57)
Region of residency				
Large cities	13.5	17.9	24.6	1 (ref.)
Middle-sized towns	17.6	22.8	28.8	1.22 (1.04–1.42)
All others	16.1	26.9	31.3	1.20 (1.03–1.40)
Daily (tobacco) smoking				
Yes	15.4	24.3	31.7	1.11 (0.98–1.27)
No (including past use)	15.9	22.1	27.5	1 (ref.)
Physical exercise				
Inactive/occasionally	20.9	27.1	33.5	1 (ref.)
At least once a week	8.4	14.5	18.4	0.72 (0.64–0.80)

Study population: 3311 participants who completed all three measurements. Odds ratios (OR) with 95 % confidence intervals (CI). Impaired-SRH (=1) was defined as self-reported health status other than good SRH (=0). The prevalence was calculated based on complete cases. The ORs and CIs were estimated by alternating logistic regression (*n* = 3311) with multiple imputations on missing data (<1%). ^1^Adjusted for sex and age (continuous). ^2^Full model, adjusted for all covariates, including sex and age (continuous). ^3^Based on an index score from a principal component analysis (including the variables: visiting cinema, theatre, concert, art museum, other museums, and sermon). The participants were approximately grouped into four quartiles based on their level of cultural attendance (the two middle quartiles were combined into one group): Low (≈25%, lowest quartile), Middle (≈50%, middle two quartiles), and High (≈25%, highest quartile). ^4^Adjusted for age only.

**Table 3.** The association between cultural participation level and impaired self-reported health (SRH), three measurements (3311 participants).

Cultural participation^1^	Model 1 OR (CI)	Model 2 OR (CI)	Model 3 OR (CI)
Low	2.39 (2.06–2.77)	2.30 (1.97–2.69)	1.91 (1.62–2.26)
Middle	1.57 (1.38–1.79)	1.61 (1.41–1.85)	1.45 (1.25–1.67)
High	1 (ref.)	1 (ref.)	1 (ref.)

Odds Ratio (or) with 95% confidence intervals (CI) by a population average model were estimated by generalized estimating equations (GEE) with multiple imputations. Data included three measurements (the participants were the same in each measurement): 1982/83, 1990/91, and 1998/99. Model 1: Crude. Model 2: Sex- and age-adjusted. Model 3: fully adjusted for all covariates (i.e. sex; age; educational level; marital status, region of residency; daily smoking; and physical exercise). [1] based on an index calculated from the scores in a principal component analysis with one factor (including the variables: visiting cinemas, theatre, concerts, art museums, other museums, and sermons). The participants were approximately grouped into four quartiles based on their level of cultural attendance (the two Middle quartiles were combined into one group): low (≈25%, lowest quartile), Middle (≈50%, middle two quartiles), and high (≈25%, highest quartile).

### Complementary analyses

The findings from the sub-analysis and the two sensitivity analyses (results not shown in tables) yielded similar findings as in the main analyses (Table 3). First, the findings from the sub-analysis (full GEE model), including two measurements only, aligned well with the ORs from our main analysis but seemed to be somewhat weaker. In the full model, compared with high cultural participation level, low cultural participation level was associated with an OR of 1.78 (95% CI, 1.47–2.16) and middle cultural partici­pation level with an OR of 1.22 (95% CI, 1.02–1.45) for Impaired-SRH. Second, the sensitivity analysis (full GEE model), not including current daily (tobacco) smoking and physical exercise in the adjustments, yielded slightly higher ORs than the full model in the main analyses; compared with high cultural participation level, low cultural participation level was associated with an OR of 2.06 (95% CI, 1.75–2.43) and middle cultural participation level with an OR of 1.50 (95% CI, 1.30–1.73) for Impaired-SRH. Third, the sensitivity analysis using ALR yielded similar associations as in the main analyses (Table 3). In the crude model, compared with high cultural participation level, low cultural participation level was associated with an OR of 2.39 (95% CI, 2.07–2.77) and middle cultural participation with an OR of 1.62 (95% CI, 1.43–1.83) for Impaired-SRH. The corresponding ORs decreased throughout adjustments. In the age and sex adjusted model, compared with high cultural participation level, low cultural participation level was associated with an OR of 2.23 (95% CI, 1.91–2.60) and middle cultural participation with an OR of 1.62 (95% CI, 1.41–1.85) for Impaired-SRH. In the full model, compared with high cultural participation level, low cultural participation level was associated with an OR of 1.86 (95% CI, 1.58–2.19) and middle cultural participation with an OR of 1.45 (1.26–1.67) for Impaired-SRH. In addition, no interactions were found between cultural participation level and each of the included covariates for Impaired-SRH (data now shown).

## Discussion

This longitudinal study was based on a random sample of the Swedish population [25], with three measurements that collected individual data on cultural participation, SRH, sociodemographic variables and lifestyle during the follow-up. The study suggested a strong association between cultural participation and SRH. The results were similar in the two sensitivity analyses and a sub-analysis including the first two measurements only. We also found indication of a gradient, suggesting a dose-response relationship in our models that were based on time-varying covariates.

The evidence supporting the use of culture-based interventions to improve public health outcomes is rapidly growing [35,36]. The findings of our study on SRH align with most previous findings on well-being and SRH^12^ [18–21,37], as well as mortality and morbidity [6–16,27,38]. The associations found in our study were also similar in direction and magnitude to those found on adolescents in Norway [18], adults in Finland [19] and Denmark [12], and in a longitudinal study (spanning over ten years) of adults 50 years or older in England [37]. Our longitudinal study extends previous evidence by showing that the effect of cultural participation on SRH seems to sustain over time and suggests a graded association between cultural participation and SRH after taking the time-varying nature of our data into account.

The mechanisms on health regarding the experience of culture in humans are complex and not entirely understood, and may involve both biological, behavioral, psychological, and social mechanisms [26]. For example, an experiment of aesthetic experiences in the Sanctuary of Vicoforte, Italy, showed stress reduction (cortisol levels) as well as increased wellbeing [21]. Experiments with exposures of live and recorded music in different age groups indicated that the emotional response to music encompassed many positive feelings such as arousal, joy, and calmness [2]^8^.These feelings could possibly be explained by modulating effects of music in brain structures involved in emotions [39] and the psychobiological stress system [40]. There are also animal experiments of the influence of a positive environment on brain functions. Laboratory mice in an ′enriched environment′ (meaning a larger cage, toys to play with, a running wheel, and company of others) had better memory, better learning, enhanced synaptic density in the brain, higher neurogenesis, and higher levels of neurotrophic proteins [41–44]. In addition, delays of Alzheimer’s and Parkinson’s diseases has also been found [42].

Our findings should be interpreted in the light of certain limitations. First, despite controlling for several potentially important confounders and use of longitudinal data with up to three measurements, unmeasured and residual confounding might still exist. For example, differences in economic factors (e.g. financial assets and wealth) may have affected the associations; however, we do not expect this potential confounding to be substantial given that we adjusted for age, sex and education. In addition, we cannot rule out potential mediating roles of some covariates (e.g. smoking status and physical activity). However, excluding these covariates from the adjustments yielded similar associations as when these covariates were included, which contradicts their potential mediating effects. Nevertheless, further studying are needed to determine the roles of these and other covariates as mediators and moderators, given the putatively complex and multifactorial causal pathways between cultural participation and health [26]. Furthermore, self-reported bias [45] is a potential limitation when using interview-based variables. Additionally, the study accounted for missing values (about 1% of some covariates) by MI and potential pitfalls that may have accompanied this approach needs to be considered. For example, we cannot be fully confident in the missing at random assumption when using this approach. However, the findings were based on established imputation approaches and analytic methods [30]. Moreover, this study did not aim to determine the ideal combination of specific types of cultural activities for the promotion of good health and wellbeing; future research could address these gaps. Future longitudinal studies could also include both active (creative) and passive (receptive) cultural participation in relation to SRH, as both have been linked with SRH [11,12]. Lastly, since our study is based on survey data from Sweden collected during the period 1982–1999, its findings should be confirmed in longitudinal studies conducted in other countries and in more recent years. For example, availability to and differences in specific types of cultural activities as well as population demographic changes might affect its generalizability. In addition, although the study included participants from a random sample of the Swedish population, we cannot rule out selection bias and be certain that the sample corresponds to the current Swedish population. For example, changes in population demographics have occurred in recent years (e.g. due to immigration) [46], which have resulted in a larger proportion of residents originating from other countries and regions that can have different cultural behaviors. Therefore, future studies could consider potential differences in cultural activities within the population over time and the effect of these on SRH. Moreover, several of the previous studies have been conducted in culturally similar settings (e.g. European countries) [12,18,19,23,24,37], meriting studies in settings where cultural behavior and activities may be different. However, our dataset did not include ethnicity.

Nevertheless, there were several important strengths in this study which balances out its limitations. The main strength was the use of longitudinally collected data. Furthermore, the consistency of our results with those from other studies on cultural participation and various health outcomes [6–9,18–21,27,37,38] supports the validity of our findings. Although the present study was unable to determine causality, several features of the present study supports a possible causal association between cultural participation and health, including the longitudinal nature of the data, the use of time-varying covariates and imputation for missing values [30], the observed dose-response relationship, the consistency between older and more recent studies in humans [6–9,15,16,18–21,27,37,38,47], the coherence with animal data [41–44], and plausibility suggestions [2,48]. Given that SRH is a valid epidemiological measurement that has been shown to predict mortality [22], accessibility to cultural activities in the society seems to be an important factor for promoting improved health and longevity.

In conclusion, this showed a graded longitudinal association between cultural participation and SRH, with low cultural participation being associated with Impaired-SRH. The evidence collected hitherto, together with our findings, suggests that engagement in cultural activities during leisure time may promote health. This insight could be particularly valuable for policymakers and primary healthcare providers seeking to enhance public access to cultural activities and encourage patients to participate in them, which may improve both individual well-being and public health.

## Data Availability

This study made use of national registers and, owing to legal concerns, data cannot be made openly available. Further information regarding the registry used in this study is available from *Statistics Sweden* (SCB): https://www.scb.se/en/; ULF: https://www.scb.se/en/finding-statistics/statistics-by-subject-area/living-conditions/living-conditions/living-conditions-surveys-ulfsilc/.

## References

[1] Grossi E. Beauty and health: an intriguing liaison? Clin Exp Rheumatol. 2024;42(5):1091–1096. doi: 10.55563/clinexprheumatol/65340f. 38451159

[2] Dewey J. Art as Experience. (Capricorn Books, New York.; 1958.

[3] Drehmer JE, Hipple B, Ossip DJ, et al. A Cross-Sectional Study of Happiness and Smoking Cessation among Parents. J Smok Cessat. 2017;12(1):6–14. doi: 10.1017/jsc.2015.6. PMC5289385 28163788

[4] Södergren M, Sundquist J, Johansson S-E, et al. Physical activity, exercise and self-rated health: a population-based study from Sweden. BMC Public Health. 2008;8:352. doi: 10.1186/1471-2458-8-352. PMC2576235 18840294

[5] CULTURE FOR HEALTH. https://www.cultureforhealth.eu. Last. accessed ; 2023-09-08.

[6] Bygren LO, Konlaan BB, Johansson SE. Attendance at cultural events, reading books or periodicals, and making music or singing in a choir as determinants for survival: swedish interview survey of living conditions. BMJ. 1996;313(7072):1577–1580. doi: 10.1136/bmj.313.7072.1577. PMC2359094 8990990

[7] Løkken BI, Merom D, Sund ER, et al. Cultural participation and all-cause mortality, with possible gender differences: an 8-year follow-up in the HUNT Study, Norway. J Epidemiol Community Health. 2020;74(8):624–630. doi: 10.1136/jech-2019-213313. 32303593

[8] Fancourt D, Steptoe A. The art of life and death: 14 year follow-up analyses of associations between arts engagement and mortality in the English Longitudinal Study of Ageing. BMJ. 2019;367:l6377. doi: 10.1136/bmj.l6377. PMC7190042 31852659

[9] Bygren LO, Jansåker F, Sundquist K, et al. Association between attending cultural events and all-cause mortality: a longitudinal study with three measurements (1982-2017). BMJ Open. 2023;13(2):e065714. doi: 10.1136/bmjopen-2022-065714. PMC9945101 36810171

[10] Fancourt D, Steptoe A. Physical and Psychosocial Factors in the Prevention of Chronic Pain in Older Age. J Pain. 2018;19(12):1385–1391. doi: 10.1016/j.jpain.2018.06.001. PMC6288062 29949780

[11] Cuypers K, Krokstad S, Holmen TL, et al. Patterns of receptive and creative cultural activities and their association with perceived health, anxiety, depression and satisfaction with life among adults: the HUNT study, Norway. J Epidemiol Community Health. 2012;66(8):698–703. doi: 10.1136/jech.2010.113571. 21609946

[12] Jensen A, Folker AP, Lindström M, et al. Arts and culture engagement for health: a Danish population-based study. Public Health. 2023;225:120–126. doi: 10.1016/j.puhe.2023.09.012. 37925836

[13] Johansson S-E, Jansåker F, Sundquist K, et al. A longitudinal study of the association between attending cultural events and coronary heart disease. Commun Med (Lond). 2023;3(1):72. doi: 10.1038/s43856-023-00301-0. PMC10209104 37225790

[14] Løkken BI, Merom D, Sund ER, et al. Association of engagement in cultural activities with cause-specific mortality determined through an eight-year follow up: the HUNT Study, Norway. PLoS One. 2021;16(3):e0248332. doi: 10.1371/journal.pone.0248332. PMC7951895 33705441

[15] Jensen A, Pirouzifard M, Lindström M. Arts and culture engagement and mortality: a population-based prospective cohort study. Scand J Public Health. 2024;52(4):511–520. doi: 10.1177/14034948231165853. PMC11179309 37086102

[16] Nenonen T, Kouvonen A, Härkänen T, et al. Cultural activities and all-cause mortality among Finnish adults: a 19-year follow-up. BMC Public Health. 2025;25(1):1906. doi: 10.1186/s12889-025-23032-4. PMC12100814 40410697

[17] Løkken BI, Sund ER, Krokstad S, et al. Association between engagement in cultural activities and use of general practitioner consultations: 7-year follow-up of adults from the HUNT study, Norway. BMJ Open. 2023;13(9):e068004. doi: 10.1136/bmjopen-2022-068004. PMC10496689 37696637

[18] Hansen E, Sund E, Skjei Knudtsen M, et al. Cultural activity participation and associations with self-perceived health, life-satisfaction and mental health: the Young HUNT Study, Norway. BMC Public Health. 2015;15:544. doi: 10.1186/s12889-015-1873-4. PMC4460785 26055410

[19] Nenonen T, Kaikkonen R, Murto J, et al. Cultural services and activities: the association with self-rated health and quality of life. Arts & Health. 2014;6(3):235–253. doi: 10.1080/17533015.2014.897958.

[20] Johansson SE, Konlaan BB, Bygren LO. Sustaining habits of attending cultural events and maintenance of health: a longitudinal study. Health Promot Int. 2001;16(3):229–234. doi: 10.1093/heapro/16.3.229. 11509458

[21] Grossi E, Tavano Blessi G, Sacco PL. Magic Moments: determinants of Stress Relief and Subjective Wellbeing from Visiting a Cultural Heritage Site. Cult Med Psychiatry. 2019;43(1):4–24. doi: 10.1007/s11013-018-9593-8. 29998384

[22] Lorem G, Cook S, Leon DA, et al. Self-reported health as a predictor of mortality: a cohort study of its relation to other health measurements and observation time. Sci Rep. 2020;10(1):4886. doi: 10.1038/s41598-020-61603-0. PMC7078209 32184429

[23] Weziak-Bialowolska D, Bialowolski P. Cultural events - does attendance improve health? Evidence from a Polish longitudinal study. BMC Public Health. 2016;16:730. doi: 10.1186/s12889-016-3433-y. PMC4974712 27495252

[24] Węziak-Białowolska D. Attendance of cultural events and involvement with the arts—impact evaluation on health and well-being from a Swiss household panel survey. Public Health. 2016;139:161–169. doi: 10.1016/j.puhe.2016.06.028. 27491889

[25] SCB. Statistics Sweden. (In Swedish: Statistikmyndigheten) Statistics on Income and Living Conditions. (In Sweidsh: Undersökningarna av levnadsförhållanden [ULF]). Statistisk Centralbyrån, SCB. LE0101 (5120). http://www.scb.se/le0101 or http://www.scb.se/le0101-en. [Latest accessed 2022-02-09]. ; 2021.

[26] Fancourt D, Aughterson H, Finn S, et al. How leisure activities affect health: a narrative review and multi-level theoretical framework of mechanisms of action. Lancet Psychiatry. 2021;8(4):329–339. doi: 10.1016/S2215-0366(20)30384-9. PMC7613155 33581775

[27] Lutgendorf SK, Russell D, Ullrich P, et al. Religious participation, interleukin-6, and mortality in older adults. Health Psychol. 2004;23(5):465–475. doi: 10.1037/0278-6133.23.5.465. 15367066

[28] Theorell T, Bojner Horwitz E. Emotional Effects of Live and Recorded Music in Various Audiences and Listening Situations. Medicines (Basel). 2019;6(1). doi: 10.3390/medicines6010016. PMC6473529 30678173

[29] Morrone A. Guidelines for measuring cultural participation. ; 2006. UNESCO Institute for Statistics. © UNESCO-UIS. UIS/TD/06-02. ISBN: 978-92-9189-059-0. http://www.uis.unesco.org/. [Accessed 2022-10-31].

[30] Engels JM, Diehr P. Imputation of missing longitudinal data: a comparison of methods. J Clin Epidemiol. 2003;56(10):968–976. doi: 10.1016/s0895-4356(03)00170-7. 14568628

[31] StataCorp. Stata 19 Multiple-Imputation Reference Manual. College Station, TX: stata Press ; 2025.

[32] Hardin JW. in Encyclopedia of Statistics in Behavioral Science. ; 2005.

[33] Carey V, Zeger SL, Diggle P. Modelling Multivariate Binary Data with Alternating Logistic Regressions. Biometrika. 1993;80(3):517–526. doi: 10.2307/2337173.

[34] Bland JM, Altman DG. Multiple significance tests: the Bonferroni method. BMJ. 1995;310(6973):170. doi: 10.1136/bmj.310.6973.170. PMC2548561 7833759

[35] Stuckey HL, Nobel J. The connection between art, healing, and public health: a review of current literature. Am J Public Health. 2010;100(2):254–263. doi: 10.2105/AJPH.2008.156497. PMC2804629 20019311

[36] Rodriguez Novo N, Novo Munoz MM, Cuellar-Pompa L, et al. Trends in Research on Art Therapy Indexed in the Web of Science: a Bibliometric Analysis. Front Psychol. 2021;12:752026. doi: 10.3389/fpsyg.2021.752026. PMC8639497 34867642

[37] Tymoszuk U, Perkins R, Spiro N, et al. Longitudinal Associations Between Short-Term, Repeated, and Sustained Arts Engagement and Well-Being Outcomes in Older Adults. J Gerontol B Psychol Sci Soc Sci. 2020;75(7):1609–1619. doi: 10.1093/geronb/gbz085. PMC7424284 31287550

[38] Bygren LO, Johansson S-E, Konlaan BB, et al. Attending cultural events and cancer mortality: a Swedish cohort study. Arts & Health. 2009;1(1):64–73. doi: 10.1080/17533010802528058.

[39] Koelsch S. Brain correlates of music-evoked emotions. Nat Rev Neurosci. 2014;15(3):170–180. doi: 10.1038/nrn3666. 24552785

[40] Thoma MV, La Marca R, Brönnimann R, et al. The effect of music on the human stress response. PLoS One. 2013;8(8):e70156. doi: 10.1371/journal.pone.0070156. PMC3734071 23940541

[41] Benito E, Kerimoglu C, Ramachandran B, et al. RNA-Dependent Intergenerational Inheritance of Enhanced Synaptic Plasticity after Environmental Enrichment. Cell Rep. 2018;23(2):546–554. doi: 10.1016/j.celrep.2018.03.059. PMC5912949 29642011

[42] Bygren LO. Intergenerational health responses to adverse and enriched environments. Annu Rev Public Health. 2013;34:49–60. doi: 10.1146/annurev-publhealth-031912-114419. 23297658

[43] Schrijver NC, Pallier PN, Brown VJ, et al. Double dissociation of social and environmental stimulation on spatial learning and reversal learning in rats. Behav Brain Res. 2004;152(2):307–314. doi: 10.1016/j.bbr.2003.10.016. 15196798

[44] Kempermann G. Environmental enrichment, new neurons and the neurobiology of individuality. Nat Rev Neurosci. 2019;20(4):235–245. doi: 10.1038/s41583-019-0120-x. 30723309

[45] Althubaiti A. Information bias in health research: definition, pitfalls, and adjustment methods. J Multidiscip Healthc. 2016;9:211–217. doi: 10.2147/JMDH.S104807. PMC4862344 27217764

[46] Tønnessen M, Aradhya S, Mussino E. How Assad changed population growth in Sweden and Norway: syrian refugees’ impact on Nordic national and municipal demography. PLoS One. 2021;16(1):e0244670. doi: 10.1371/journal.pone.0244670. PMC7816981 33471846

[47] Jensen A, Thygesen LC, Folker AP, et al. Arts and culture engagement for health: a Danish population-based follow-up study. Scand J Public Health. 2025. 14034948251399932. doi: 10.1177/14034948251399932. 41404980

[48] Caldwell LL. Leisure and health: why is leisure therapeutic? British Journal of Guidance & Counselling. 2005;33(1):7–26. doi: 10.1080/03069880412331335939.

